# Dopamine Treatment and Cognitive Functioning in Individuals with Parkinson's Disease: The “Cognitive Flexibility” Hypothesis Seems to Work

**DOI:** 10.1155/2014/260896

**Published:** 2014-01-30

**Authors:** Alberto Costa, Antonella Peppe, Ilenia Mazzù, Mariachiara Longarzo, Carlo Caltagirone, Giovanni A. Carlesimo

**Affiliations:** ^1^I.R.C.C.S. Santa Lucia Foundation, Via Ardeatina 306, 00179 Rome, Italy; ^2^Clinical Neurology, University of Rome, “Tor Vergata”, Via Orazio Raimondo 18, 00173 Rome, Italy

## Abstract

*Background*. Previous data suggest that (i) dopamine modulates the ability to implement nonroutine schemata and update operations (flexibility processes) and that (ii) dopamine-related improvement may be related to baseline dopamine levels in target pathways (inverted U-shaped hypothesis). *Objective*. To investigate above hypotheses in individuals with Parkinson's disease (PD). *Methods*. Twenty PD patients were administered tasks varying as to flexibility load in two treatment conditions: (i) “off” condition, about 18 hours after dopamine dose and (ii) “on” condition, after dopamine administration. PD patients were separated into two groups: low performers (i.e., performance on Digit Span Backward below the sample mean) and high performers (i.e., performance above the mean). Twenty healthy individuals performed the tasks in two sessions without taking drugs. *Results*. Passing from the “off” to the “on” state, only low performer PD patients significantly improved their performance on high-flexibility measures (interference condition of the Stroop test; *P* < 0.05); no significant effect was found on low-flexibility tasks. *Conclusions*. These findings document that high-flexibility processes are sensitive to dopamine neuromodulation in the early phases of PD. This is in line with the hypothesis that striatal dopamine pathways, affected early by PD, are precociously implicated in the expression of cognitive disorders in these individuals.

## 1. Introduction

Dopamine is a brain catecholamine originating from subcortical neurons. It supplements the activity of several neural circuitries belonging to both subcortical and neocortical structures. At this level, dopamine acts as a neuromodulator of synapses involved in the mediation of various aspects of human functioning and behaviour [1]. The role of dopamine in complex mental operations is being increasingly recognized thanks to the results of studies in both healthy individuals and clinical populations [2–5].

A focus has been posed on the study of dopamine neuromodulation of mental operations underpinned by the activity of prefrontal neurons. Indeed, the prefrontal cortex has abundant dopamine receptors and receives important dopamine fibre projections from the striatum and ventral-tegmental regions [6]. In fact, working memory, set-shifting, planning, and selective attention abilities, all of which are considered to critically depend on the integrity of the prefrontal-striatal loops [7], have been shown to be particularly sensitive to dopamine activity [8]. Nevertheless, the results of available studies have failed to show a linear effect of dopamine stimulation on human cognition; indeed, they alternatively document a positive (cognitive improvement) or a negative (cognitive worsening) effect. Cools et al. [2] documented that the administration of bromocriptine (a D2 family agonist) had a significant effect on the cognitive performance of healthy subjects as a function of their working memory capacity (considered the indirect expression of the functioning of dedicated brain areas). More specifically, the authors found significant improvement on a task tapping updating abilities that was restricted to the experimental subgroup, which obtained the lowest working memory score in a baseline evaluation [2]. This finding is congruent with other evidence from genetic studies in both human [9, 10] and nonhuman samples [11, 12] and suggests that the effect of dopamine stimulation on cognitive prefrontal functions is inversely correlated with the baseline dopamine level in target neural networks (U-shape inverted hypothesis) [1, 13]. In other words, a subject presenting with reduced ability to perform a dopamine-dependent task, and, thus, believed to have low functioning dopamine transmission in dedicated brain pathways, would benefit from dopamine replacement more than a subject who performed normally on the same test who, differently, would be detrimentally affected by taking dopamine.

This hypothesis is also supported by data from Parkinson's disease (PD) patients. In fact, PD is characterized early by a deficit of dopamine brain pathways due to the primary loss of dopamine neurons in the substantia nigra and ventral tegmental area; the nigrostriatal dopamine circuitry would be precociously affected by the disease, which subsequently also injures the mesocortical and mesolimbic pathways [14, 15]. In a previous study, pergolide (D1 + D2 family agonist) and pramipexole (D2 + D3 family agonist) administration was shown to significantly improve updating abilities (i.e., *n*-back working memory performance) in a subgroup of PD subjects who exhibited the lowest working memory baseline scores [16]. By contrast, when the PD patients with the highest baseline working memory scores took the pharmacological compounds, their performance scores were unaffected.

Nevertheless, some other findings indicate that the variability of the effect of dopamine supplementation on cognition cannot be fully explained by the assumptions of the inverted U-shaped hypothesis. In fact, in PD patients dopamine-related cognitive improvement on working memory and prospective memory tasks was detected without differentiating between low and high performers [17, 18]. Moreover, Kimberg and D'Esposito [19] found that administration of pergolide to healthy individuals improved performance of the subgroup with the highest baseline working memory scores but not that of the subgroup with the lowest baseline performance. To interpret these contrasting data, some authors argued that the effect of dopamine can be differentially modulated by tasks that require the implementation of cognitive processes, allowing for the flexible manipulation and updating of the information/mental representations (namely, cognitive flexibility processes) versus tests sensitive to the ability to maintain the mental representations stable over time (i.e., cognitive stability processes) [1, 8]. The equilibrium between these two sets of mental operations, that is, integrated utilization of cognitive flexibility and cognitive stability processes, is necessary for the optimal adaptation of the individual to his environment, and it should be sustained by the synergic and complementary activity of phasic and tonic Dn receptors belonging to the D2 and D1 family, respectively. In particular, phasic D2 activity should allow the flexible modification of mental representations by signalling the need for nonroutine schemata to be implemented, whereas tonic D1 activity should mediate the capacity to retain stable representations in the face of incoming information [20]. The distribution pattern of Dn receptors in the brain makes the striatum a suitable candidate to represent the neural substrate for flexible behaviour; conversely, dopamine activity in the prefrontal cortex seems to have a critical role in supporting continuous maintenance of stable mental operations [21, 22]. Indeed, D2 receptors are more numerous in the striatal regions than in the prefrontal cortex where, instead, D1 receptors are represented to a greater extent [23, 24].

The hypothesis that the effect of dopamine on cognitive functioning depends on the regional distribution of Dn receptors mediating flexibility/stability processes is based on some animal and humans findings. Studies from nonhuman primates reveal that the inhibition of dopamine neurotransmission produces opposite effects on cognition depending on whether it is applied at the level of the prefrontal cortex or the striatum. In particular, prefrontal cortex dopamine lesions have been shown to improve attentional set-shifting, whereas these same abilities have been found impaired after lesions over the striatum [25]. Healthy human volunteers were negatively affected by a switching task after taking sulpiride (a D2 antagonist), whereas their performance on a task requiring the maintenance of stable mental representations actually improved after taking the drug [5]. Cools et al. [2] documented a differential effect of bromocriptine administration on the activity of the prefrontal and striatal neurons in healthy individuals. In fact, after the subjects took bromocriptine, enhanced striatal activity was found during flexible updating, whereas the same potentiation activity was observed in the prefrontal cortex when the need to strengthen the maintenance of mental representations was stressed by the task.

Results of studies investigating the relationship between dopamine and cognitive functioning in persons suffering from PD provide some relevant clues for understanding the effect of dopamine on the modulation of cognitive flexibility/stability processes. As stated above, the core pathology underlying PD is the degeneration of dopamine cells in the midbrain that leads to precocious and severe dopamine depletion in the striatum, where D2 receptors are particularly abundant [23, 24]. More specifically, dopamine depletion in PD primarily involves the rostrodorsal extent of the head of the caudate nucleus and only later affects the ventral tegmental neurons projecting to more ventral parts of this structure and to prefrontal and limbic regions [14, 15]. According to the view that striatal activity is particularly related to cognitive flexibility processes [7, 26], in the early stages of PD dopamine stimulation should primarily modulate performance on tasks requiring flexible behaviour. Indeed, several studies have reported that PD patients perform worse than healthy controls on tasks investigating updating, highly demanding working memory abilities and prospective memory [8, 27, 28]. Studies directly assessing the effect of dopamine withdrawal/administration on cognition in PD are also in line with the idea that dopamine compounds improve patients' ability to implement flexibility operations without affecting their capacity to maintain stable memories [18, 29, 30].

Summarizing the above discussion, two main hypotheses are currently advanced to explain the effect of dopamine on frontal-like functions in humans: (i) dopamine modulates performance in a dissociable way as a function of the flexibility load of the task; (ii) dopamine action is inversely correlated with the baseline dopamine level in target networks. The aim of the present study was to verify these hypotheses in individuals without dementia in the early stages of PD. With this in mind, we contrasted the effect of dopamine administration/withdrawal on the performance of two groups of PD patients, with discrepant basal cognitive performance (i.e., low versus high performers) on tasks sensitive to the integrity of dopamine dependent pathways and on tasks with relatively low and high flexibility loads. According to the above hypotheses, we predict that (i) dopamine compound administration improves performance on high flexibility load tasks and that (ii) this improvement should be particularly observed in PD individuals with lower baseline performance scores.

## 2. Materials and Methods

### 2.1. Subjects

Twenty individuals with idiopathic PD (11 female and 9 male) and 20 normal controls (NC; 9 female and 11 male) participated in this study after giving their informed consent. Human data included in this paper was obtained in compliance with the Helsinki Declaration. The clinical characteristics of the PD group and the sociodemographic characteristics of both groups are reported in Table 1. The diagnosis of idiopathic PD was made according to the United Kingdom Parkinson's Disease Society Brain Bank Criteria [31]. Exclusion Criteria: (i) disease duration >5 years; (ii) dementia based on Diagnostic and Statistical Manual of Mental Disorders Criteria for dementia (American Psychiatric Association, 1994) and a Mini Mental State Examination Score <25 [32, 33]. PD patients were also administered the Unified Parkinson's Disease Rating Scale-Part III (UPDRS) [34].

All but two patients were administered dopamine therapy with levodopa, and nine of them also received cotherapy with a dopamine-agonist or IMAO-B; two patients received only dopamine agonists. The mean dosage of levodopa equivalent [35] was 336 mg (SD = 150). PD patients were administered a battery of neuropsychological tests to assess a wide range of cognitive functions (see below for a description of the neuropsychological battery). The neuropsychological assessment was carried out with patients under regular dopamine therapy in one session performed two weeks to one month before the experimental sessions began.

NC individuals were recruited from PD patients' relatives. Exclusion criteria for the subjects in the NC group were head trauma, history of psychiatric disease, central nervous system diseases, severe systemic disease, and taking drugs that can affect cognitive performance.

### 2.2. Neuropsychological Test Battery

We assessed the following cognitive domains: episodic memory (Immediate and delayed recall of a 15-word list [36]; immediate and delayed prose recall; Carlesimo et al., 2002; Rey's figure form A reproduction [37]), short-term memory (Corsi Block Tapping test. [38]; Digit Span Forward; [38]), executive functions (Modified Card Sorting test [39]), language (Naming subtest of the Aachener Aphasia test [40]), abstract reasoning (Raven's Coloured Progressive Matrices [36]), and constructive praxis (Copy of Drawings and Copy of Drawings with Landmarks [36]). Published normative data for score adjustment according to age, education, and gender as well as normality cutoff scores (corresponding to a performance ≥95% of the lower tolerance limit of the normal population distribution) were available.

### 2.3. General Design

PD patients were evaluated after one month of full dosage, stable dopamine treatment. They were assessed in two experimental conditions performed on different days, with an intersession interval of about one month. In one condition, PD subjects performed the experimental tasks in the morning after 18/24 h of drug withdrawal (“Off” condition; CAPIT [41]). In the other condition, they were examined 90–120 minutes after the first morning administration of levodopa and/or dopamine agonists, considered as their best “on” condition. NCs performed the experimental tasks in two different sessions, with an intersession interval of about one month, called “blue” and “green.” The tests were given in the morning without taking drugs. The “blue” session was associated with the off session and the “green” one with the on session. The order of the experimental conditions (off/blue versus on/green) was randomized across subjects.

### 2.4. Experimental Cognitive Battery

#### 2.4.1. Digit Span Backward

In this task [38], subjects are instructed to repeat strings of digits (varying in length from 3 to 9 digits) read by the examiner in the reversed order. The score is the longest sequence correctly reproduced (score range: 0–9). Based on performance on this task, patients and NCs were classified as low and high performers.

#### 2.4.2. Stroop Test

The Stroop test [42] is commonly used to assess executive deficits in neurological populations [43]. It includes three subtests, each consisting of a sheet paper displaying 100 stimuli regularly aligned in ten columns and ten rows. The subject has to scrutinize the sheet paper, as quickly as possible, beginning at the left upper board and proceeding vertically down to the tenth column on the right. In the first subtest (word reading subtest), the subject has to read five colour-name words (red, blue, green, brown, and violet). In the second subtest (naming colours subtest), the subject is presented with coloured squares (i.e., above colours) and is asked to name their colours. In the third subtest, written words representing the above colours are shown in conflicting colours (e.g., the word red written with blue ink) and the subject has to name the ink colour of the printed word but not read the word (resistance to interference subtest). To evaluate performance, response times (i.e., the time needed to complete the subtest) and accuracy (i.e., sum of subject's correct answers) are recorded for each subtest.

#### 2.4.3. Verbal Fluency Tasks

For this test, two parallel forms were constructed and their order of administration was randomized across the two experimental therapy conditions and across subjects. Each form consists of three subtests: (i) phonemic [36], (ii) semantic [44], and (iii) alternate phonemic/semantic fluency [45, 46]. On the phonemic subtest, the subject is requested to generate words beginning with the letters “A,” “F,” and “S” (or, with the letters “C,” “E,” and “L,” on the parallel form) in three different trials, each lasting 60 seconds. In the semantic subtest, the subject is asked to say words belonging to the “colours,” “animals” and “fruits” categories (or, “threes,” “furniture,” and “cities,” in the parallel form) in three different trials, which also lasted 60 seconds each. The alternate phonemic/semantic task is an extradimensional shifting task [45, 46] in which subjects have to continuously alternate words beginning with a specific letter with words belonging to a particular category as follows: trial (1) letter “A” and “colours”; trial (2) letter “F” and “Animals”; trial (3) letter “S” and “Fruits” (letter “C” and “Threes”; letter “E” and “furniture”; letter “L” and “Cities,” in the three trials of the parallel form, resp.). Three trials were given, each lasting 60 sec. At the beginning of each fluency task, a training trial was given to be sure the subject understood the rules of the task. The number of words correctly generated within 60 sec. was recorded.

### 2.5. Complementary Measures

Previous findings in PD patients have shown an association between on/off states, fluctuations of mood and anxiety symptoms [47], and a potential negative influence of affective disorders on cognitive performance [48, 49]. Therefore, the State and Trait Anxiety Inventory-State Anxiety (STAY-S) [50] and the Beck Depression Inventory (BDI) [51, 52] were administered to both PD and healthy participants in the two experimental sessions.

## 3. Results

### 3.1. PD Patients' Performance on the Tests of the Neuropsychological Battery

PD patients' average scores and number (and percentage) of patients performing below the normality cut-off score on the tests of the neuropsychological battery are reported in Table 2. Nine patients showed dysexecutive deficits (pathological scores on the Modified Card Sorting test), two had constructive praxis impairment (pathological scores on the Copy of Drawings test), and one patient had long-term memory disorders (pathological scores on Delayed Recall of the 15-Word List).

### 3.2. Effect of Dopamine Treatment on PD Patients' Performance on Experimental Cognitive Tasks

To evaluate the hypothesis that the effect of dopaminergic compounds on cognitive performance in the PD group varied as a function of basal performance level on a task of prefrontal cortex activity (i.e., low performers have the greatest improvement after dopamine treatment), according to Kimberg and D'Esposito [19] suggestions PD patients were split based on their average accuracy on the Digit Span Backwards. To classify patients as “high” or “low” performers, we chose to use the average score obtained in both the “off” and “on” therapy conditions. This was done in order to rule out potential confounds due to score regression toward the mean effect [3]. Therefore, PD patients who obtained an accuracy score (averaged across the two experimental sessions) below the mean of the whole PD group (mean value = 4.1) were classified as low performers (*n* = 13), and PD patients whose average score fell above the average of the PD group were classified as high performers (*n* = 7). The on therapy condition was the first assessment session for eight low performers and three high performers.

Preliminary one-way ANOVAs showed that high and low performer PD patients did not significantly differ in years of formal education (mean = 12.4; SD = 3.8 and mean = 9.1; SD = 4.8, resp.; *F*(1,18) = 2.40; *P* > .10), age (mean = 70.1; SD = 5.3 and mean = 68.1; SD = 8.7, resp.; *F*(1,18) = 0.32; *P* > .50), disease duration (mean = 3.2; SD = 1.9 and mean = 2.2; SD = 1.9, resp.; *F*(1,18) = 1.15; *P* > .20), and MMSE score (mean = 27.6; SD = 2.3 and mean = 27.6; SD = 1.7, resp.; *F*(1,18) = .00; *P* > .90). A two-way ANOVA also revealed that dopamine administration improved significantly UPDRS scores (*F*(1,18) = 19.2; *P* < .001). The lack of the group effect (*F*(1,18) = 2.29; *P* > .10) and the group*Treatment interaction (*F*(1,18) = 1.88; *P* > .10) confirmed that this effect was comparable in the two PD subgroups (high performer group: off condition: mean = 20.7; SD = 6.3; on condition: mean = 11.3; SD = 3.6. Low performer group: off condition: mean = 15.2; SD = 7.6; on condition: mean = 10.2; SD = 3.9).

The same criterion as that followed for splitting PD patients was used to classify NCs as high and low performers. So, NCs with score higher than the mean of their group on the digit span backward (i.e., average score between scores obtained in both experimental sessions) were classified as high performers (*n* = 5). Subjects with Digit Span score below the group mean have been classified as low performers (*n* = 15). Mixed ANOVAs with Clinical Group (PD patients versus NCs) and Working Memory Group (low versus high performers) as between factors, Treatment (off versus on therapy condition for PD patients; blue versus green sessions for NCs), and Trial (Subtest 1 versus Subtest 2 versus Subtest 3) as within factors were then performed. In the case of analyses regarding the scores on the Stroop test, the Trial factor was defined by word reading versus naming colours versus resistance to interference subtests scores. As for statistical analyses involving fluency tasks, the Trial factor was defined by phonemic versus semantic versus alternate phonemic/semantic fluency subtests scores.

#### 3.2.1. Stroop Test

**Figure 1:**
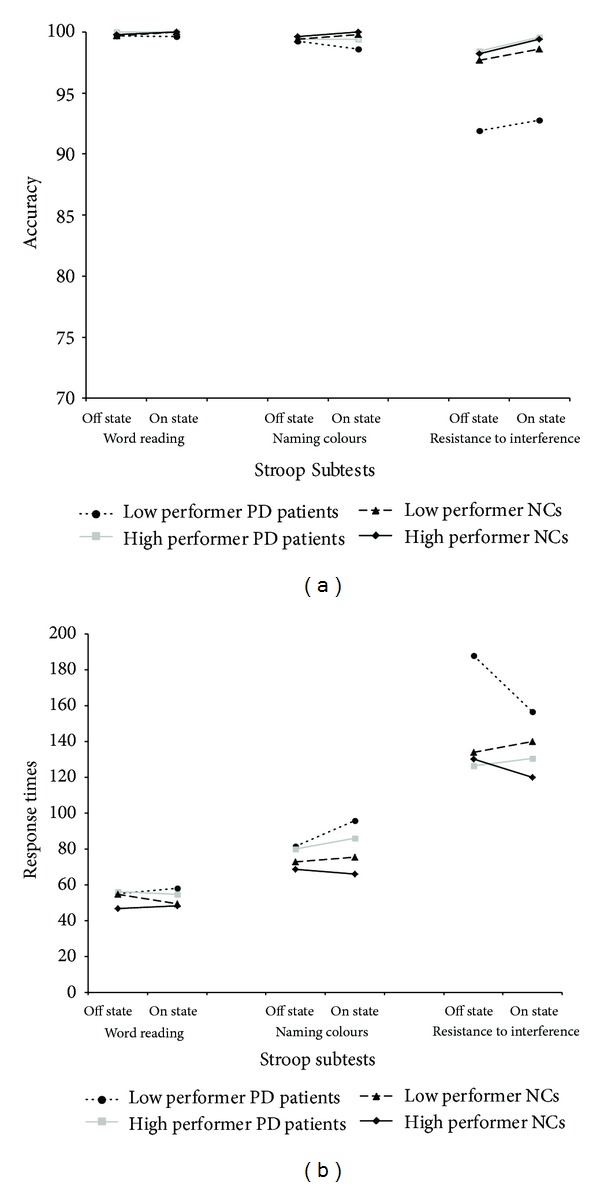
Average performance on Stroop subtests of individuals in the four experimental groups. Accuracy (a) and response times (b) are reported.

Subjects' scores on this test are reported in Table 3 and illustrated in Figure 1.

*Accuracy.* The only effect to reach the statistical significance was that of the main factor Trial (word reading versus naming colours versus resistance to interference subtests scores; (*F*(2,72) = 4.30; *P* = .017). Tukey HSD tests showed that subjects in both experimental groups were significantly less accurate in the resistance to interference subtest (mean = 97.1; SD = 4.8) compared to both the word reading (mean = 99.8; SD = .42; *P* = .01) and the naming colours (mean = 99.4; SD = 0.98; *P* = .03) subtests. However, no significant differences between the two latter subtests were found (*P* > .80). No other significant effects were found (all *P* consistently >.10).

*Response Times.* Also in this case, the main effect of Trial reached statistical significance (*F*(2,72) = 109.7; *P* < .001), whereas the effect of Clinical Group (PD versus NCs) approached significance (*F*(3,36) = 3.06; *P* = .097). The first level Working Memory Group*Trial interaction approached the statistical significance (*F*(2,72) = 2.77; *P* = .069), whereas the third level Clinical Group*Working Memory Group*Treatment*Trial interaction was fully significant (*F*(2,72) = 5.09; *P* = .008). Tukey HSD tests showed that low performer PD patients significantly reduced their response times passing from the off to the on condition only on the resistance to interference subtest (*P* = .002); no significant between-sessions difference was found for the high performer PD patients and both NC subgroups (in all cases *P* > .10). Moreover, while in the off therapy condition, average response latency of low performer PD patients on the resistance to interference subtest was longer than that of the other three experimental groups (*P* < .001 in all cases), in the on therapy condition their performance was found to be worse only than that exhibited by high performer NCs (*P* = .032). In fact, their performance in on therapy condition was fully comparable to that exhibited by high performer PD patients and by low performer NCs (*P* > .10 in both cases). No other effect involving the Clinical Group and the Working Memory Group resulted to be significant (all *P* consistently >.10).

#### 3.2.2. Fluency Tasks

**Figure 2:**
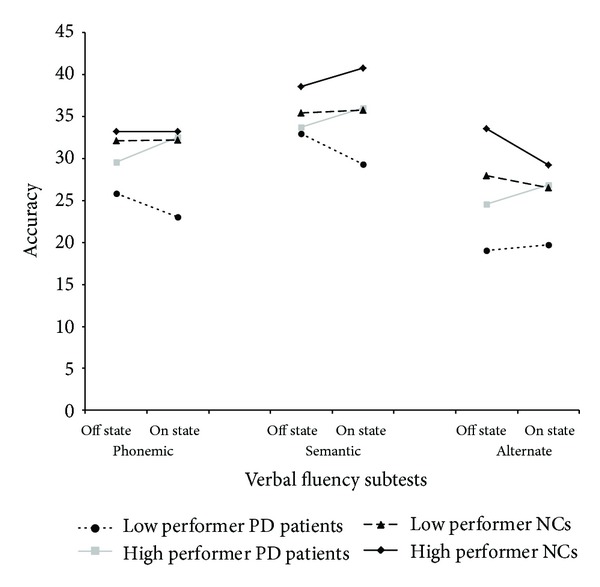
Average performance on fluency subtests of individuals in the four experimental groups on fluency subtests.

Subjects' scores on this test are reported in Table 3 and illustrated in Figure 2.

The effects of the main factors of Clinical Group (*F*(1,36) = 6.93; *P* = .012), Working Memory Group (*F*(1,36) = 4.35; *P* = .044), and Trial (*F*(2,72) = 20.65; *P* < .001) were significant, whereas the effect of Treatment was not (*F*(1,36) = 0.01; *P* > .80). No interaction reached statistical significance (all *P* consistently >.10). Tukey HSD analyses revealed that PD patients generated fewer words (mean = 27.8; SD = 8.9) than NCs (mean = 33.1; SD = 7.4; *P* = .006) and that in both PD and NC groups low performers (mean = 28.3; SD = 9.5) tended to be worse than high performers (mean = 32.6; SD = 6.7; *P* = .083). Post hoc comparisons made to qualify the effect of Trials showed that, in all subjects, average accuracy score on the alternate phonemic/semantic task (mean = 25.9; SD = 10.3) was lower than that on both the phonemic (mean = 30.2; SD = 9.9; *P* = .005) and semantic (mean = 35.3; SD = 8.6; *P* < .001) tasks. Average accuracy on the phonemic task, in turn, was lower than that observed on the semantic task (*P* < .001).

*Relationship between Working Memory Scores and Cognitive Performance Changes between the Two Experimental Conditions.* Splitting the PD sample in two small subgroups could affect the power of statistical analyses, thus limiting possible inferences from data. To further verify the existence of a significant relationship between working memory performance (Digit Span Backward scores) and the effect of dopamine withdrawal/administration, we applied a linear regression model (stepwise) to data from the whole PD sample. In particular, separate regression models were performed for each of the different cognitive tests administered in the off and on conditions. In each model the dependent variable was the rate of performance difference between the two experimental conditions on that specific measure according to the following formula: [(on performance-off performance)/off performance]; in all cases the explicative factors were the digit span backward score and the order of administration of the experimental conditions. The model was significant only when applied to the performance changes on the interference condition of the Stroop test (response times). In this case, the variable entering the regression equation was the score subjects achieved on the Digit Span Backward (*F*(1,18) = 5.06; *β* = 0.47; *P* = .037), while the order of experimental conditions administration did not (*β* = 0.01; *P* > .90). This indicates that, in PD patients, lower scores on the Digit Span Backward significantly predict response times decrease on the Stroop test (i.e., interference condition) passing from the off to the on condition. Results of regression analyses involving performance changes on the other cognitive measures administered document that no independent variable enters in the equation.

#### 3.2.3. Effect of Dopamine Treatment on Complementary Measures Scores

To investigate the effect of dopamine administration/withdrawal on the Beck Depression Inventory and STAY-S scores of PD patients, two ANOVAs with Clinical Group (PD patients versus NCs) and Working Memory Group (low versus high performers) as between factors, Treatment (off versus on therapy condition for PD patients; blue versus green sessions for NCs) as within factor, were performed.

Beck Depression Inventory Score. The absence of the main effects of Clinical Group (*F*(1,36) = 2.96; *P* > .09), Working Memory Group (*F*(1,36) = 2.82; *P* > .10), Treatment (*F*(1,36) = 0.22; *P* > .10), and the interaction between the main factors (all *P* consistently >.09) show that depressive symptoms are comparably severe in low performer PD patients (mean = 10.6; SD = 5.9), high performer (mean = 8.3; SD = 7.2) PD patients, low performer NCs (mean = 8.9; SD = 8.1), and in high performer NCs (mean = 3.2; SD = 2.6), and that they do not vary as a function of the dopamine therapy condition in the PD group (off state: mean = 9.9; SD = 6.7; on state: mean = 9.0; SD = 6.4).

STAY-S Score. In this case, the Clinical Group*Working Memory Group Interaction was significant (*F*(1,36) = 4.34; *P* = .0044). However, Tukey HSD tests performed to qualify this interaction failed to evidence significant between groups difference (all *P* consistently >.10). This indicates that, although high performer NCs tended to show the lowest STAY-S scores (mean = 33.7; SD = 8.0), these appear not to be significantly different from those obtained by low performer PD patients (mean = 40.4; SD = 6.0), high performer PD patients (mean = 46.7; SD = 14.3), and by low performer NCs (mean = 41.3; SD = 12.5). Moreover, the absence of significant interactions involving the factor Treatment (all *P* consistently >.10) evidences that there was no difference between off (mean = 44.9; SD = 10.6) and on (mean = 42.2; SD = 9.8) states in the PD group.

## 4. Discussion

The aim of the present study was to investigate two major hypotheses concerning the effect of dopamine therapy on cognitive functions in individuals with Parkinson's disease. The first hypothesis predicted that the ameliorative effect of dopamine would be greater on high than low cognitive flexibility tasks [1] and, the second hypothesis predicted that this effect should be greater in PD patients with lower basal cognitive performance on tasks sensitive to the integrity of dopamine dependent pathways, which are assumed to represent an indirect measure of dopamine levels in target circuitries [1]. In order to verify the two hypotheses, we contrasted the effect of dopamine administration/withdrawal on the performance of two groups of PD patients with discrepant levels of backward digit span on tasks with relatively low and high flexibility loads.

The findings of the study give support to both hypotheses. In fact, results show that, after they took dopamine, PD patients classified as low performers reduced their response times selectively on one of the high flexibility measures used, that is, the resistance to interference condition of the Stroop test; in fact, in the “on” condition their performance was comparable to that of both healthy controls (i.e., low performers) and high performers PD patients. The reduced response latency cannot simply be attributed to increased impulsivity after taking the dopamine compound, because low performer PD patients did not obtain a worse accuracy score on this task passing from the on to the off condition. Instead, in the high performer PD group no significant effect of pharmacological manipulation was found on high flexibility tasks. Above effects are confirmed by results of regression analyses performed on the PD sample taken as a whole. Indeed, these results document that lower scores on the variable here used to classify PD patients as low-and high performers (Digit Span Backward) significantly predict a greater improvement after dopamine therapy intake selectively on the Stroop test (i.e., resistance to interference condition).

Compared with the four tasks we can consider as low flexibility measures (i.e., the Stroop test: word reading and colour naming; fluency tasks: phonological and semantic fluency subtests), the interference condition of the Stroop test makes high demands on executive processes that require inhibiting the adoption of overriding automatic response to implement nonroutine schemata [46, 53, 54]. Conversely, the four low-flexibility measures tap sustained attention and require continuous checking and monitoring of mental representations and encoding strategies and working memory [46, 54], which do not stress the ability to discount more salient stimuli or override prepotent responses.

The request to inhibiting the adoption of overriding automatic response also differentiates the interference resistance subtest of the Stroop test from the alternate fluency task that just requires the implementation of shifting abilities without the need to inhibit automated processes. In fact, we found no significant effect of dopamine administration on PD patients' performance on the latter task. It could be argued that the poor performance of our low performer PD patients on the alternate fluency task might be partially related to processes different from shifting, which may be less sensitive to dopamine modulation. In fact, compared with healthy controls these patients show reduced ability to generate words also in the single phonemic and semantic fluency subtests. So, difficulty in accessing the retrieval of stored information and in checking and monitoring mental representations could affect alternate fluency accuracy without implying shifting abilities.

However, the differential effect of dopamine therapy withdrawal/administration we found on the interference resistance subtest of the Stroop test and on the alternate fluency task is congruent with results of previous PD studies that failed to evidence a significant effect of dopamine administration on shifting tasks [55, 56] and may indicate that in the early phases of PD dopamine replacement specifically improves the ability to disengage from a previously learned behaviour to choose between competing responses rather than shifting aptitude per se. This interpretation is particularly in line with findings outlining that, in a competing response paradigm, dopamine administration significantly enhanced the PD patients' ability to analyze between-stimuli incongruence [57]. In a subsequent experiment in healthy participants, these authors document that the task execution required recruitment of the striatum and, particularly, the dorsal caudate nucleus [57]. The authors argued that the dorsal striatum might be critically involved in reducing the bias produced by salient stimuli when various types of information have to be processed and integrated for a response to be selected [57]. This observation concurs with previous evidence in nonhuman primates [58, 59] and humans [26], suggesting that the (dorsal) striatum is particularly implicated in flexibility conditions that require selecting among various stimuli and competing responses.

Interestingly, it has been proposed that striatal activity, mediated by phasic D2 receptor integrity, allows the flexible modification of mental representations by signalling the need for implementation of nonroutine schemata to prefrontal neurons; conversely, prefrontal cortex activity, mediated by the action of tonic D1 receptors, allows the stable maintenance of mental representations [20, 22]. Indeed, our data could be interpreted in this vein. The dopamine-related effect here found specifically referred to the Stroop paradigm (interference modulation) that properly requires choosing between competing responses by overriding automated behaviour, previously reported to be sensitive to frontal-striatal activity also in PD subjects [26]. Moreover, based on the temporal progression of dopamine system alteration in PD, taking into consideration that the PD sample in this study included only individuals in the early stage of the disease, the hypothesis could be advanced that the ameliorative effect produced by dopamine administration on PD patients' flexibility scores observed here is due to better modulation of the activity of the frontal-(dorsal) striatal network. Indeed, dopamine depletion precociously involves the rostrodorsal extent of the head of the caudate nucleus, a region strongly connected to the dorsolateral prefrontal cortex. Only later does it affect the more ventral parts of this structure, which are preferentially connected to the ventral prefrontal cortex [14, 15, 60]. However, due to the absence of functional neuroimaging investigation further studies are needed to explore this issue.

In conclusion, although the relatively low sample size may represent a limit of the present study, our results lend support to the idea that flexibility processes allowing the adoption of nonroutine schemata in between-response competing conditions are affected early by dopamine dysregulation in PD, thus indicating that cognitive mechanisms involved in these conditions are particularly sensitive to dopamine brain stimulation. The dopamine-related effects we found cannot be explained as due to a general influence of drug administration/withdrawal on the general efficiency of attention processes; furthermore, they are not affected by some potentially confounding factors such as presence of anxiety and depressive symptoms. This information appears to be useful in a clinical perspective. Indeed, dopamine functioning is altered in several medical conditions (e.g., schizophrenia and attention deficit hyperactivity disorder). In this vein, an integrated therapeutic approach that takes into account the potential effect of dopamine drug manipulation on cognitive functions would likely allow better management of the disease [27].

## Figures and Tables

**Table 1:** Sociodemographic and clinical characteristics of participants in the PD and control groups.

	Individuals with PD	Normal controls	*F* (1, 38)	*P* level
Gender (F/M)	11/9	9/11		
	Mean (SD)			
Age (years)	68.8 (7.6)	65.5 (8.0)	1.8	>0.10
Years of formal education	10.3 (4.7)	12.1 (3.5)	1.9	>0.10
MMSE^a^ score	27.6 (1.8; range: 25–30)	29.4 (1.4; range: 25–30)	11.6	<0.01
UPDRS^b^ score (off state)	16.6 (7.5)			
Years of disease duration	2.43 (1.9; range 0.08–5)			
Age at disease onset	66.4 (7.4)			

^a^MMSE: Mini Mental State Examination; ^b^UPDRS: Unified Parkinson's Disease Rating Scale.

**Table 2:** PD patients' performance (average scores and percentage of pathological values) on the test of the neuropsychological battery. For all tests, with the exception of Modified Card Sorting test-perseverative and nonperseverative errors, higher scores mean better performance.

Neuropsychological tests	PD group *N* = 20	PD patients with pathological score
	Mean (SD)	*N* (percentage)
Mnesic functions		
*Short-term memory *		—
Corsi Test Forward	4.8 (0.6)	—
Digit Span Forward	5.6 (1.5)	—
*Episodic memory *		1 (5%)
Word list recall-immediate recall	42.4 (9.4)	—
Word list recall-delayed recall	9.4 (2.6)	1
Word list recognition-correct items	13 (1.8)	—
Word list recognition-false recognition	1.9 (1.6)	—
Prose recall-immediate recall	5.6 (1.2)	—
Prose recall-delayed recall	5.7 (1.3)	—
Rey's figure-immediate reproduction	14.1 (6.1)	
Rey's figure-delayed reproduction	12.7 (6.9)	
Visual-spatial abilities		2 (10%)
Free hand copying of drawings	9.1 (1.9)	2
Copying drawings with landmarks	67.3 (2.4)	—
Rey's figure copy	30.3 (4.9)	—
Language abilities		1 (5%)
Object naming	28.5 (1.8)	—
Abstract reasoning		
Raven's progressive matrices 47	27.8 (4.1)	—
Executive functions		9 (45%)
Modified Card Sorting Test-categories achieved	4.5 (1.3)	9
Modified Card Sorting test-perseverative errors	7.4 (5.9)	5
Modified Card Sorting Test-nonperseverative errors	5.9 (5.5)	4

**Table 3:** Subjects' performance (average values) on the cognitive tests administered in the two experimental sessions.

	Low performer PD patients		High performer PD patients		Low performer NCs		High performer NCs	
	Off condition	On condition	Off condition	On condition	Blue session	Green session	Blue session	Green session
	Mean (SD)							
Stroop test								
Word reading								
Response times	54.9 (13.6)	58.1 (30.3)	56.0 (15.2)	54.4 (19.9)	54.5 (21.5)	49.3 (8.7)	46.8 (9.7)	48.0 (5.9)
Accuracy	99.7 (0.6)	99.6 (0.9)	100 (0.0)	100 (0.0)	99.7 (1.3)	100.0 (0.0)	99.8 (0.5)	100 (0.0)
Naming colours								
Response times	81.5 (18.4)	95.7 (48.9)	79.7 (12.9)	86.0 (22.9)	72.8 (20.5)	75.4 (16.8)	68.4 (17.4)	65.8 (16.4)
Accuracy	99.2 (1.2)	99.6 (2.4)	99.4 (0.9)	99.4 (0.8)	99.4 (1.1)	99.8 (0.4)	99.6 (0.9)	100 (0.0)
Resistance to interference								
Response times	187.6 (57.8)	156.3 (55.0)	126.1 (37.7)	130.4 (53.2)	133.9 (44.5)	139.9 (44.5)	130.2 (53.1)	120.4 (27.3)
Accuracy	91.9 (12.9)	92.8 (13.2)	98.4 (1.5)	99.6 (0.8)	97.7 (5.2)	98.6 (2.3)	98.2 (1.9)	99.4 (0.9)
Fluency tests								
Phonemic	25.8 (11.9)	23.0 (8.7)	29.6 (9.5)	32.3 (9.0)	32.1 (11.1)	32.2 (8.1)	33.2 (4.2)	33.2 (6.9)
Semantic	33.0 (9.6)	29.3 (8.6)	33.7 (4.5)	36.0 (9.3)	35.4 (11.7)	35.8 (5.6)	38.6 (3.4)	40.8 (1.6)
Alternate phonemic/semantic	19.1 (9.0)	19.7 (8.3)	24.6 (7.6)	26.8 (11.2)	28.0 (12.7)	26.5 (9.6)	33.6 (8.0)	29.2 (6.1)
